# A Comparison of Collection Techniques for Gene Expression Analysis of Human Oral Taste Tissue

**DOI:** 10.1371/journal.pone.0152157

**Published:** 2016-03-24

**Authors:** Nicholas Steven Archer, Dongli Liu, Jan Shaw, Garry Hannan, Konsta Duesing, Russell Keast

**Affiliations:** 1 Food and Nutrition, CSIRO, Sydney, Australia; 2 Centre for Advanced Sensory Science, School of Exercise and Nutrition, Deakin University, Melbourne, Australia; The University of Tokyo, JAPAN

## Abstract

Variability in human taste perception is associated with both genetic and environmental factors. The influence of taste receptor expression on this variability is unknown, in part, due to the difficulty in obtaining human oral tissue that enables quantitative expression measures of taste genes. In a comparison of six current techniques (Oragene RNeasy Kit, Isohelix swab, Livibrush cytobrush, tongue saliva, cheek saliva collection, and fungiform papillae biopsy), we identify the fungiform papillae biopsy is the optimal sampling technique to analyse human taste gene expression. The fungiform papillae biopsy resulted in the highest RNA integrity, enabling amplification of all the assessed taste receptor genes (*TAS1R1*, *TAS1R2*, *TAS1R3*, *SCNN1A* and *CD36*) and taste tissue marker genes (*NCAM1*, *GNAT3* and *PLCβ2*). Furthermore, quantitative expression was observed in a subset of taste genes assessed from the saliva collection techniques (cheek saliva, tongue saliva and Oragene RNA kit). These saliva collection techniques may be useful as a non-invasive alternative sampling technique to the fungiform papillae biopsy. Identification of the fungiform papillae biopsy as the optimal collection method will facilitate further research into understanding the effect of gene expression on variability in human taste perception.

## 1 Introduction

The presence of taste cells in the oral cavity is an essential component in our ability to taste the foods we consume. The taste cells express specialised receptors that sense the nutrient profile of ingested foods, with each taste cell tuned to a single taste quality i.e. sweet only, bitter only [1]. Binding of a taste ligand to its cognate receptor (or ion through a taste associated ion channel) elicits a secondary messenger cascade within taste cells signalling the taste quality of the ingested food (i.e. sweet or bitter, respectively). While we detect the sweetness of a lollypop and the bitterness of tonic water, the perceived intensity varies significantly from person to person.

It is widely acknowledged that taste thresholds and perceived intensities differ significantly between individuals, and also within an individual over time. Mounting evidence shows that genetics contributes to inter-individual variability in taste, for example, differences in the bitterness to PROP [2] and quinine [3], or sweet taste [4, 5]. Other factors are also involved, with twin studies indicating the environment may account for between 40–80% of the variability in tastes [6–9]. Environmental factors (i.e. the diet or health status of an individual) and gene x environment interactions may alter taste gene expression and the level of taste proteins/receptors produced. To clarify the role of genetics and the environment in taste physiology will require robust quantitative measures of taste gene expression.

A considerable amount of information in the understanding of human taste physiology is drawn through the use of animal models. Caution must be taken as taste is highly species specific and findings from animal based studies are not always directly relevant to humans. This is because taste is a vital sense that is under intense selective pressure finely tuned to the ecological niche of that species. This is why, for example, cats do not perceive sweetness, pandas do not detect umami and rodents differ in their perception of artificial sweeteners compared to humans [10–12]. Therefore, it is important that studies analysing human taste variation are completed in humans and caution should be taken transferring findings from other animal models to humans.

The difficulty in understanding the genetic and environmental factors that influence taste are partly due to the difficulty in obtaining human taste tissue. Current techniques are either invasive and require specialist training [13, 14], or are unclear as to whether they can provide quantitative measures of taste gene expression [15, 16]. Here we compare six previously published sampling techniques with the aim to determine which techniques will allow quantitative analysis of taste gene expression. Benefits and disadvantages are presented for the techniques which show the ability to determine taste gene expression.

## 2 Material and Methods

### 2.1 Subjects and Study Overview

Samples were collected from eight participants (5 females) with no history of any known taste disorders, on three consecutive days at 10am (2 samples/day). Participants were asked to consume their normal breakfast prior to 8am and refrain from eating and drinking (water permitted) two hours prior to collection. This research complies with the *Declaration of Helsinki* for Medical Research, all procedures were approved by the CSIRO Human Research Ethics Committee (HREC13/06), and informed written consent obtained from all participants.

**Fig 1:**
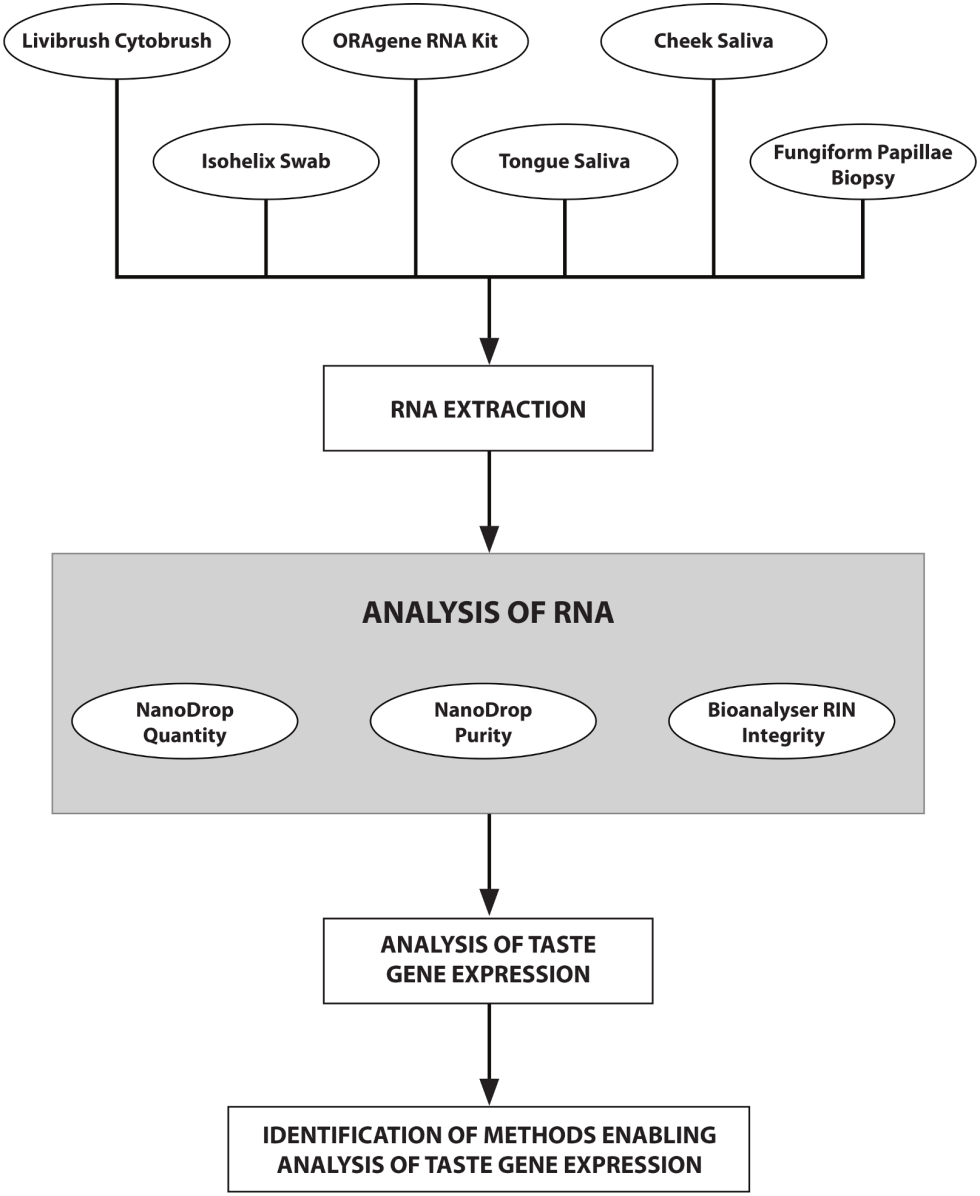
Flowchart of study design to identify collection techniques that enable quantitative measures of taste gene expression. Samples were collected from 8 volunteers using the six different methods, the RNA was extracted and analysed with the NanoDrop ND-1000 Spectrophotometer (for quantity and purity) and Bio-analyser (analysis of RNA integrity). Real-time quantitative PCR was completed on taste tissue markers and taste genes, allowing for the identification of methods enabling quantitative measures of taste gene expression.

The collection techniques analysed were selected based on previously reported methods. The fungiform papillae biopsy [14, 17, 18], tongue swab (Isohelix swab) [15, 16] and tongue and cheek saliva [19] collection techniques have been previously used to assess for expression of taste genes. The use of cytology brushes similar to the Livibrush Cytobrush have been used to assess gene expression profiles in oral cancers [20–22], while the ORAgene RNA is a commercial kit for expression analysis from saliva. Sampling of the circumvallate papillae using cup forceps was not completed as this was viewed as too invasive [13]. Fig 1 displays a flow chart of the testing procedure to compare the six collection methods.

### 2.2 Collection of Samples and RNA Extraction

All surfaces and equipment were treated with RNaseZap RNase Decontamination Wipes (Ambion, Life Technologies) prior to collection of samples or extraction of RNA.

#### 2.2.1 Livibrush cytobrush

A cytobrush with snappable stem (Livibrush, Livingstone International, Australia) was firmly rubbed over the anterior region of the tongue 5 times, turned and rubbed another 5 times. The brush was immediately placed into 500μl RNALater (Life Technologies, USA) in a 2ml centrifuge tube, agitated to dislodge the cells and the handle snapped off. The tube containing the cytobrush was centrifuged (2000rcf, 1min), the brush was discarded and the sample stored at -80°C. For extraction, samples were thawed on ice, an equal volume of phosphate buffered saline (PBS) pH 7.2 (Gibco, Life Technologies, USA) added, mixed well and centrifuged (2000rcf, 5min). The supernatant was discarded and 1ml TRIzol (Life Technologies, USA) added to the cell pellet.

#### 2.2.2 Isohelix swab

A tongue depressor was used to hold the tongue and the Isohelix swab (Cell Projects, United Kingdom) was rubbed firmly over the surface of the tongue 5 times, turned and rubbed another 5 times. The swab was immediately transferred to 500μl RNALater in the Spin+Collect cap (Cell Projects) and agitated. A 2ml centrifuge tube was placed on the Spin+Collect cap, inverted and centrifuged (13000rpm, 1min), the swab and cap were discarded and the sample stored at -80°C. For extraction, samples were thawed on ice, an equal volume of PBS added, mixed well and centrifuged (2000rcf, 5min). The supernatant was discarded and 1ml TRIzol (Life Technologies, USA) added to the cell pellet.

#### 2.2.3 ORAgene RNA kit

The ORAgene RNA collection kit (DNA Genotek, Canada) is a commercially available kit designed for the isolation of RNA from saliva. Participants were instructed to scrape their teeth over the surface of the tongue and saliva collected following the kit instructions. RNA was extracted from 250μl of the sample following the manufacture’s protocol with the Qiagen RNeasy Micro Kit (protocol ID: PD-PR-021).

#### 2.2.4 Tongue and cheek saliva samples

Tongue and cheek saliva samples were collected using an in-house developed method (unpublished data). Participants were instructed to gently scrape their teeth back and forth across the front of the tongue or gently scrape the teeth over the cheeks and expectorate the saliva into a 15ml centrifuge tube (total 2ml). Centrifuge tubes were kept on ice during the collection procedure (2-10min) and were snap frozen in dry ice and stored at -80°C. To extract the RNA, samples were thawed on ice and 2ml of PBS added and mixed well to dilute the salivary mucins. The samples were then centrifuged (850rcf, 5min), the supernatant discarded and 1ml of TRIzol added to the samples.

#### 2.2.5 Fungiform papillae biopsy

Fungiform papillae biopsies were collected with Castroviejo curved dissecting micro-scissors (Livingstone International, Australia). Papillae were collected without anaesthetic by a qualified doctor following the technique of Spielman et al. [14] with minor modifications. Blue food colouring (diluted 1:20 with pure water) was applied to the tongue immediately prior to the biopsy to aid in the identification of fungiform papillae. Fungiform papillae were transferred immediately to PBS and then to 500μl RNALater (Life Technologies, USA). All samples were stored at -80°C. Participants were contacted a week following papillae biopsy to ensure no adverse effects from the procedure.

A trial was undertaken to determine the appropriate sample homogenisation method for papillae biopsy samples (S1 File). Samples were thawed on ice and all six collected papillae were used for comparison with the other collection techniques. For the bead mill, eight 2.3mm Zirconia/silica beads and approximately twenty-five 1mm Zirconia/silica beads (Biospec Products, USA), 1ml of ice cold TRIzol and thawed tissue sample were added to a 2ml centrifuge tube. Samples were homogenised using a Retsch Mixer Mill MM300 for 4 minutes at 30/s. For the pellet pestle, tissue samples were transferred to a 2ml U-bottom shaped centrifuge tube containing 150μl Trizol and homogenised with Kimble chase cordless motor pellet pestle (Sigma-Aldrich, USA) for 3-4min or until the sample was completely dispersed. Additional TRIzol was added for a final volume of 1ml and the sample was passed through a 19 gauge needle 15 times to ensure complete lysis of cells. The pellet pestle was superior to the bead mill for RNA extraction from papillae biopsy (S1 File), and therefore, was the method used to homogenise fungiform papillae biopsy prior to TRIzol extraction.

#### 2.2.6 TRIzol extraction and sodium acetate reprecipitation

After the addition of 1ml TRIzol, samples were passed through a 19 gauge needle 15 times to ensure complete cell lysis and the RNA was extracted following the manufacturers protocol. Samples were further purified by adding sodium acetate (final concentration 0.3M) and 2.5 volumes of 100% ethanol to RNA and stored -20°C overnight. Samples were centrifuged (18000rcf, 30min at 4°C), the pellet washed with ice-cold 75% ethanol and centrifuged (18000rcf, 15min at 4°C). The supernatant was discarded and RNA resuspended in RNase free H_2_O and stored at -80°C.

### 2.3 Analysis of RNA Quantity, Purity and Quality

Samples were assessed for RNA quantity using NanoDrop ND-1000 Spectrophotometer, with the purity determined by absorbance ratio measurement at 260nm/280nm (ideal ratio of 2.0). Sample quality was determined from the RNA Integrity Number (RIN) on the Agilent 2100 Bioanalyser using either the Agilent RNA 6000 Nano Kit (samples greater than 25ng/μl) or Agilent RNA 6000 Pico Kit (samples less than 25ng/μl).

### 2.4 Gene Expression Analysis

Taste gene expression was determined from extracted RNA following standard procedures. cDNA was prepared using the High Capacity cDNA Reverse Transcription Kit (Applied Biosystems, USA) and the expression of taste genes was quantified using Taqman gene expression assays (Table 1) with all assays primers and probe located over exon junctions and spanning introns to ensure contaminating genomic DNA was not amplified. Real-time PCR was completed in 384-well plates on the Lightcycler 480 Real-time PCR Instrument (Roche, Germany). For each sample, cDNA was diluted 1 in 10 and analysed by real-time PCR in replicates of four. All experiments contained negative controls (no template control and reverse transcriptase negative control) and positive controls (RNA isolated from whole blood and gastro-intestinal tract). All Taqman assays resulted in no amplification in negative controls.

**Table 1:** Taste genes analysed by quantitative real-time PCR (Taqman assay).

Gene	Protein	Function	Taqman Assay
***PLCβ2***	PLCβ2	Signalling molecule, Type II taste marker	Hs01080542_m1
***GNAT3***	α-gustducin	Signalling molecule, Type II taste marker	Hs01385403_m1
***NCAM1***	NCAM1	Type III taste marker	Hs00941821_m1
***TAS1R1***	T1R1	Umami taste receptor	Hs00602668_m1
***TAS1R2***	T1R2	Sweet taste receptor	Hs01027711_m1
***TAS1R3***	T1R3	Sweet/umami taste receptor	Hs01026531_g1
***SCNN1A***	ENaC	α-subunit of salt taste receptor	Hs01013028_m1
***CD36***	CD36	Hypothesised fatty acid taste receptor	Hs01567185_m1
***RPLP0***	60S acidic ribosomal protein P0	Reference gene (ribosomal protein)	Hs99999902_m1
***GAPDH***	GAPDH	Reference gene (enzyme)	Hs02758991_g1
***18S rRNA***	18S rRNA	Reference gene (ribosomal RNA)	Hs99999901_s1

An aliquot of all samples cDNA (undiluted) was pooled and then serially diluted 1 in 5 to produce a standard curve. Standard curves were generated for every experiment (crossing point against log[transcript number]) and used to determine (i) the amplification efficiency of the PCR (efficiency = 10^−1/m^, where m is the gradient of the standard curve), and, (ii) to determine the relative transcript number of each sample taking into account the amplification efficiency (transcript number = 10^(Cp-b)/a^, where Cp, a and b represent the crossing point, amplification efficiency and y-intercept of the standard curve, respectively). The mean of the sample replicates was determined and the mean transcript number was normalised to three reference genes (Table 1) to determine the normalised relative transcript number for each sample.

## 3 Results

### 3.1 Study Overview

Multiple collection methods for the analysis of taste gene expression have been reported [13–19]. The aim of the study was to identify the collection technique(s) that allow robust measures of quantitative taste gene expression. Six different previously reported collection techniques were used to collect samples from 8 volunteers (Fig 1). The quality and quantity of the extracted RNA and the ability to amplify taste genes was assessed.

### 3.2 Analysis of RNA following RNA Extraction

The Livibrush cytobrush and Isohelix swab collection methods resulted in a low RNA yield and low purity as determined using the NanoDrop ND-1000 Spectrophotometer (Table 2). The fungiform papillae biopsy, tongue saliva, cheek saliva and ORAgene RNA techniques enabled a high RNA yield and high purity (Table 2). The papillae biopsy produced the highest RNA integrity (Table 2), with a RIN average greater than 8, with the other collection techniques resulting in lower quality RNA (see S1 Fig for representative Bio-analyser profiles).

**Table 2:** Comparison of RNA quantity, purity and quality from the different collection techniques.

RNA Collection Technique	Quantity: Yield μg Mean (Range)^a^	Purity: A260/280 Mean (Range)^a^	Integrity: RIN Mean (Range)^b^
**Livibrush Cytobrush**	**0.194** (0.077–0.335)	**1.42** (1.11–1.62)	**2.49** (2.30–2.70)
**Isohelix Swab**	**0.227** (0.090–0.369)	**1.66** (1.00–2.22)	**3.10** (2.10–5.20)
**Tongue Saliva**	**6.433** (0.180–15.843)	**1.85** (1.28–2.13)	**3.93** (3.40–5.80)
**Cheek Saliva**	**5.665** (0.668–15.494)	**1.99** (1.94–2.15)	**2.91** (1.10–3.90)
**ORAgene RNA Kit**	**3.324** (0.391–6.391)	**1.84** (1.75–1.96)	**5.50** (2.30–7.80)
**Fungiform Papillae Biopsy**	**4.268** (0.443–7.663)	**1.92** (1.76–2.00)	**8.14** (6.50–9.80)

^a^Nano-drop 1000 determination

^b^Bio-analyser RIN score of RNA quality (0 = poor, 10 = best)

### 3.3 Analysis of Taste Markers and Receptors for Quantitative Gene Expression

**Fig 2:**
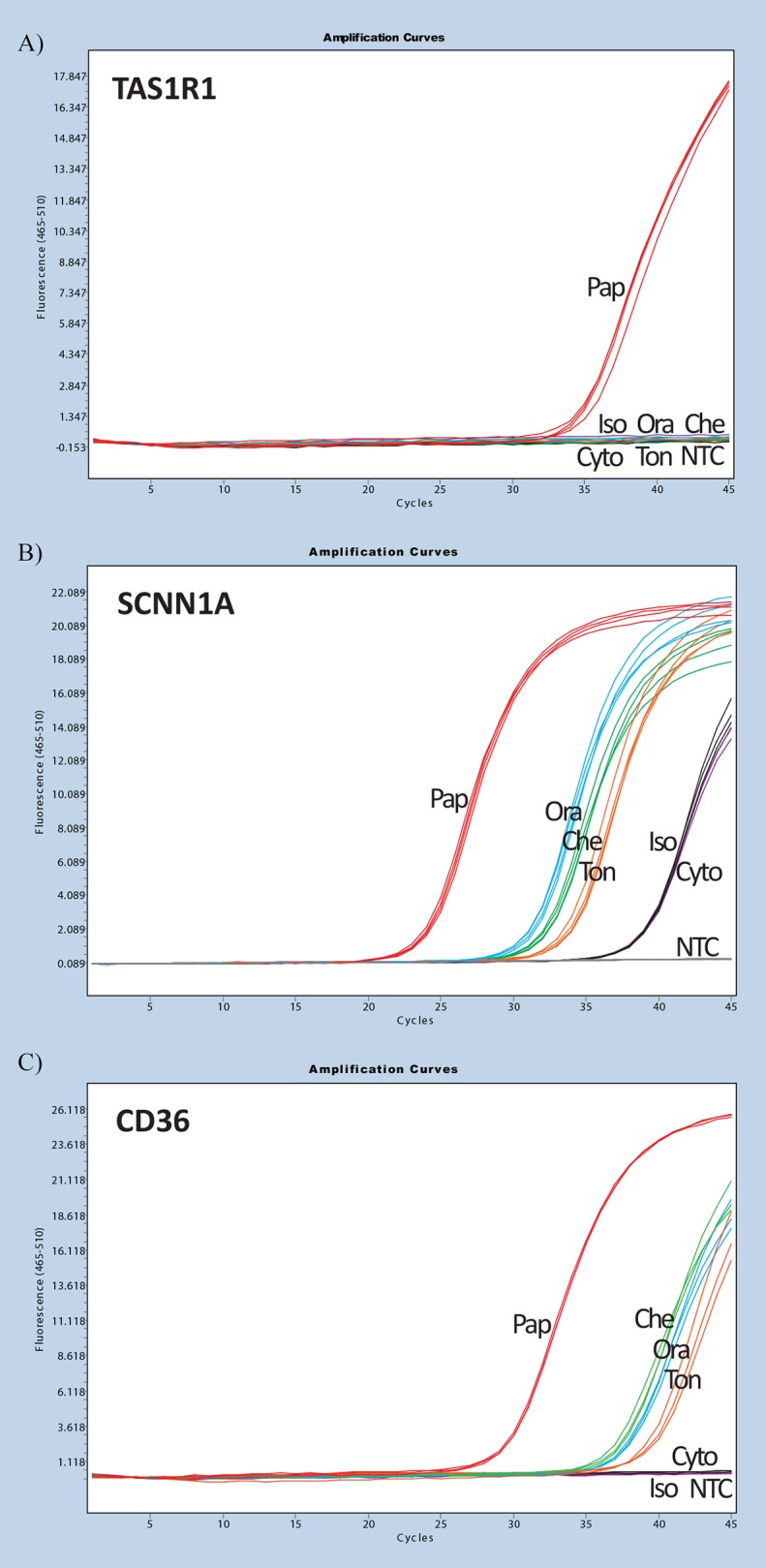
Representative Lightcycler 480 amplification profiles of taste genes using the different collection techniques. Pap—Papillae biopsy (red), Che—Cheek saliva (Green), Ton—Tongue saliva (orange), Ora—Oragene kit (blue), Iso—Isohelix brush (black), Cyto—Livibrush cytobrush (purple), NTC—no template control (grey).

The papillae biopsy was the only collection method that enabled amplification of all taste markers and taste receptors (Table 3). In particular, the papillae biopsy technique was the only one that enabled amplification for taste markers *GNAT3* (α-gustducin) and *NCAM1*; and for taste genes *TAS1R1* and *TAS1R2*. The Oragene RNA kit, tongue saliva and cheek saliva methods were able to detect a limited number of taste genes assessed and resulted in higher expression for marker *PLCβ2* (Table 3). However, the Livibrush cytobrush and Isohelix swab methods did not enable quantitative measures of taste gene expression of any of the taste genes assessed. Fig 2 shows representative amplification curves of the different collection techniques for several taste receptors.

**Table 3:** Quantitative real-time PCR analysis of taste markers and receptors using the different collection techniques^a^.

Gene	Cytobrush	Isohelix Swab	Oragene kit	Tongue saliva	Cheek saliva	Papillae biopsy
***PLCβ2***	ND	ND	32.8 (13.8)	41.3 (14.2)	59.8 (15.8)	16.6 (5.8)
***GNAT3***	ND	ND	ND	ND	ND	2.3 (1.1)
***NCAM1***	ND	ND	ND	ND	ND	8.4 (2.1)
***TAS1R1***	ND	ND	ND	ND	ND	18.2 (8.3)
***TAS1R2***	ND	ND	ND	ND	ND	✓^b^
***TAS1R3***	ND	ND	ND	ND	✓^b^	✓^b^
***SCNN1A***	0.01 (0.001)	0.01 (0.006)	1.8 (1.2)	0.7 (0.1)	1.2 (0.2)	157.9 (33.3)
***CD36***	ND	ND	1.6 (1.0)	0.1 (0.06)	8.1 (7.8)	329.3 (116.9)

^a^ Numbers represent relative transcript number for comparison between collection methods for the individual taste genes (SEM). The relative transcript number was determined (mean of 4 replicates) averaged from the 8 individuals. Grey boxes designate the inability to determine quantitative measures of gene expression. ND: not detected, expression levels were below the detection threshold in our experiment.

^b^ **✓** designates the ability to amplify gene using the sampling technique, however, the standard curve did not permit determination of transcript number (see S2 Fig for representative amplification curves).

## 4 Discussion

Our findings clearly show the fungiform papillae biopsy is the superior collection method for the analysis of quantitative taste gene expression. The papillae biopsy technique allowed for the detection of all taste genes analysed and resulted in the highest RNA integrity. A RIN score of greater than 8 is a commonly used threshold of RNA quality for use with transcriptome wide (analysis of all RNA within a population of cells) genomics techniques like microarrays and next-generation sequencing methods. Therefore, the papillae biopsy is the only technique to provide a high enough RNA quality for use with transcriptome wide genomic techniques (Table 4). The fungiform papillae collection method described by Spielman and co-workers [14] has been used to assess the presence of RNA taste transcripts in several small studies [23–26]. This technique, however, may not be ideal for all studies due to the cost associated with sample collection and the invasiveness of the procedure that may deter study participants.

**Table 4:** Comparison of collection techniques (advantages and disadvantages) for the analysis of quantitative taste gene expression.

Factor	Papillae biopsy [14,17,18]	Cheek saliva and Tongue Saliva [19]	Oragene RNA kit
**Sample quantity**	High	High	High
**Sample quality**	High (RIN value>8)	Low (RIN value<8)	Low (RIN value<8)
**Sample heterogeneity**	Heterogeneity per papillae may be high (0–9 tastebuds/papillae) [27], therefore pooling of multiple fungiform papillae recommended. Sample represents a mixture of human cells.	Homogenous sample (large collection area), however, RNA represents a mixture of human cells and oral microbial biota will constitute significant portion of sample.	Homogenous sample (large collection area), however, RNA represents a mixture of human cells and oral microbial biota will constitute significant portion of sample.
**Applications**	qPCR of all taste genes, transcriptome analysis (microarray or next generation sequencing) and histochemistry.	qPCR of pre-tested taste genes.	qPCR of pre-tested taste genes.
**Invasiveness**	Invasive (may deter study participants).	Non-invasive (amenable for adults & children).	Non-invasive (amenable for adults & children).
**Infrastructure required**	Trained doctor/dentist, dental chair, surgical equipment (i.e. micro-dissection scissors and forceps).	None—Sample snap frozen and stored at -80°C to prevent sample degradation.	None—Sample is stable at room temperature for extended time (ideal for home collection and mailing).
**Collection Cost**^a^	High (~$80/participant)^b^	Low (~$2/participant)	Medium (~$20/participant)
**RNA composition**	Mixed human RNA from taste tissue, epithelial cells, muscle and connective tissue.	Mixed RNA from human epithelial cells, human taste cells and oral micro biota.	Mixed RNA from human epithelial cells, human taste cells and oral micro biota.
**Sample size possible**	Small (n<100, due to need for trained professional and specialist equipment).	Large (n = 100–1000).	Large (n = 100–1000).

^a^Approximate cost should only be used as a guide ($AUD) and is based on the cost of sample collection only (does not include additional costs for consumables for sample extraction and storage).

^b^Cost based on fees for a doctor and surgical equipment.

While the fungiform papillae biopsy was identified as the best method for the analysis of taste gene expression, the saliva collection techniques (tongue saliva, cheek saliva and ORAgene RNA kit) may also be used to assess quantitative measures of taste gene expression from a subset of taste genes, however, pre-testing of candidate genes is recommended to ensure that quantitative measures are achievable (Table 4). Surprisingly, we identified higher *PLCβ2* expression in the tongue and cheek saliva methods compared to the papillae biopsy. This higher expression may be due to the larger size of the sampling area for these saliva methods compared to the 6–8 fungiform papillae collected for the papillae biopsy where there is likely to be significant heterogeneity between each papillae [27]. Alternatively, the observed expression may originate from another cell type also collected in the sampling technique. For example, immune cells are reported to be present in saliva and also express PLCβ2 [28,29]. Thus immune cells may the source of the higher expression observed. Each of the techniques collects a range of different cell types (Table 3), and observed expression levels represent the combined expression in the sample. Thus, caution must be taken when interpreting gene expression results (as with any mixed sample).

In contrast to the fungiform papillae biopsy, the saliva collection techniques are amenable to the collection of large cohorts as they are non-invasive, have low collection costs and are easy to collect. Table 4 compares the collection methods suitable for quantitative measures of taste gene expression to aid selection of the most appropriate method. With an understanding of the application and the expected output, the correct sampling technique can be selected to provide optimal results. While fungiform papillae biopsy and saliva techniques were successful, we identified that the Livibrush cytobrush and Isohelix swab collection techniques were unable to measure taste gene expression and we recommend these methods are not suitable for quantitative analysis of taste gene expression.

While we have identified methods enabling quantitative measures of taste gene expression, there is a need for the refinement of qPCR methods of taste tissue to ensure gene expression measures are accurate and robust. Firstly, there is a requirement for the identification of appropriate reference genes to normalise target gene expression. A growing number of studies suggest the use of several tissue specific experimentally determined reference genes for the normalisation of target gene expression, and further, recommend against using ‘housekeeping’ genes (i.e. GAPDH) for normalisation [28–30]. Therefore, identification of a validated set of taste tissue specific reference genes will enable improved measures of gene expression. Secondly, understanding the sample composition from the different collection methods is important to understand how much taste tissue is present. As highlighted above, all of the sampling procedures analysed in the current study collect a mixture of different cell types. For example, in addition to taste cells collected, the fungiform papillae also consist of epithelial and connective tissue cells and the saliva collection techniques contain oral microbes. Purification of taste cells from papillae by incubation with enzymes provides a way to enrich samples to assess only taste cells [18]. However, this is not ideal due to the length of time to extract the taste cells which likely results in a significant alteration of the gene expression profile. Therefore, identification of appropriate reference genes and understanding the composition of the sample will enable refinement of the qPCR method to ensure gene expression measures are accurate and robust.

Here we identify that the fungiform papillae biopsy is the optimal collection method for quantitative measures of taste gene expression. Recent studies provide evidence that quantitative measures of taste gene expression may explain variation in taste [19, 25]. The ability to measure quantitative gene expression in these studies is an enhancement to the majority of previous studies that test, in a binomial fashion, for the presence or absence of genes to understand taste physiology [15, 16, 23, 24]. The results will facilitate further research into understanding variability in human taste perception and the influence on food choice and preferences.

## Supporting Information

S1 Fig Representative Bio-analyser profiles from the 6 collection methods. (PDF)

S2 Fig Amplification curves of TAS1R2 and TAS1R3. (PDF)

S1 File Method and results of sample homogenisation of papillae biopsy samples. (DOCX)
