# Supplementary figures and images for: A Comparison of Collection Techniques for Gene Expression Analysis of Human Oral Taste Tissue
Source: PLoS One. 2016 Mar 24;11(3):e0152157. doi: 10.1371/journal.pone.0152157 (PMC4807031; doi:10.1371/journal.pone.0152157)

Supplementary Figure 1 - Representative Bioanalyser Results

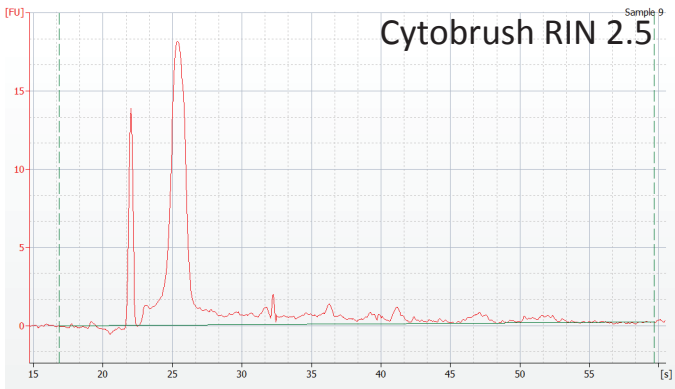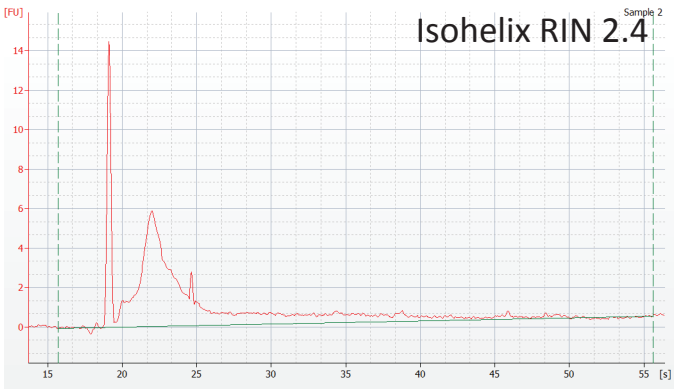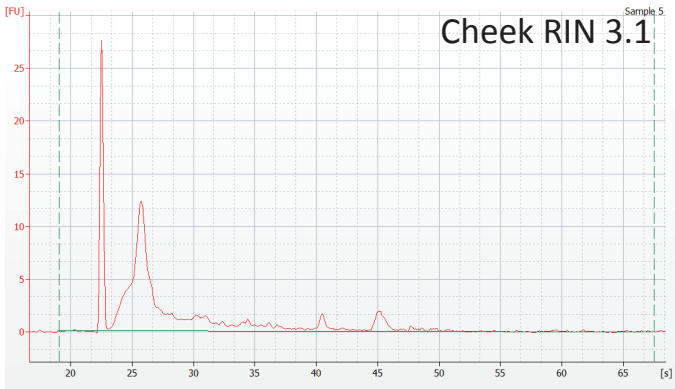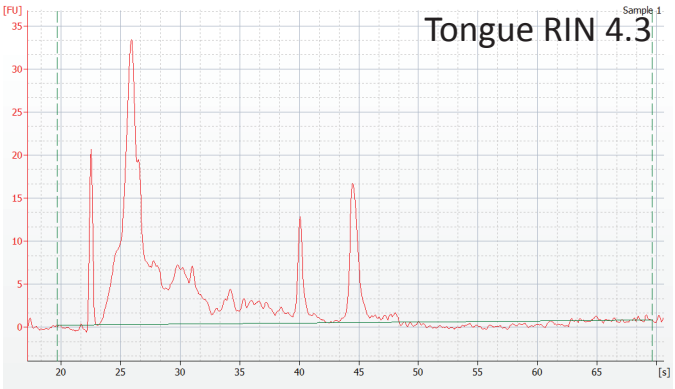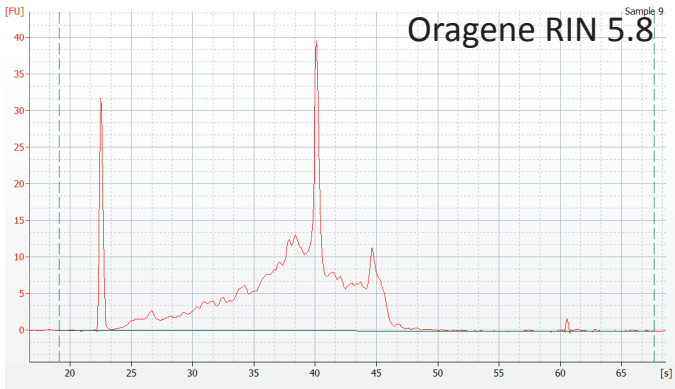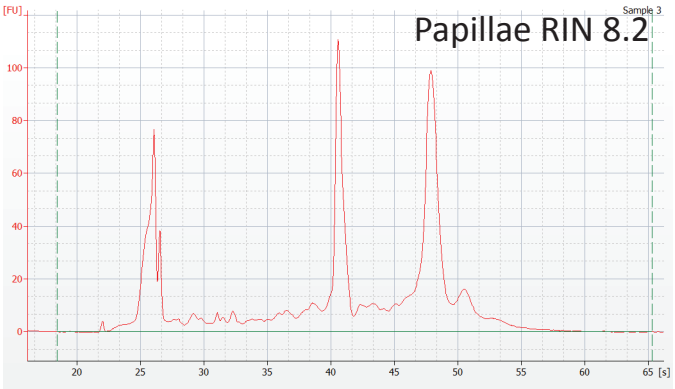

Supplement: S1 Fig — (PDF) [file pone.0152157.s001.pdf]

Supplementary Figure 2 - Amplification curves for TAS1R2 and TAS1R3

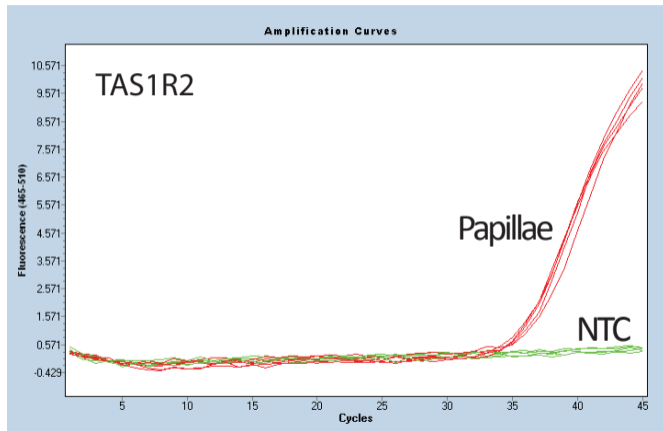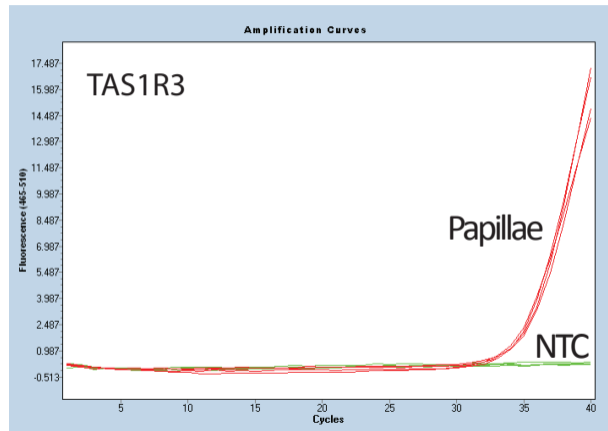

Supplement: S2 Fig — (PDF) [file pone.0152157.s002.pdf]
