# Supplementary material for: A Comparison of Collection Techniques for Gene Expression Analysis of Human Oral Taste Tissue
Source: PLoS One. 2016 Mar 24;11(3):e0152157. doi: 10.1371/journal.pone.0152157 (PMC4807031; doi:10.1371/journal.pone.0152157)
Supplement: S1 File — (DOCX) [file pone.0152157.s003.docx]

## **Supplementary file** – Comparison of homogenisation techniques for the extraction of RNA from human fungiform papillae

A trial was completed to identify an appropriate homogenisation method for the extraction of RNA from fungiform papillae. The pellet pestle has been previously used to extract RNA from fungiform papillae [25], while the bead mill allows for quick homogenisation of multiple samples at the same time. RNA was extracted from eight fungiform papillae isolated from the same individual. Each homogenisation technique was assessed with two papillae in duplicate. Fungiform papillae were thawed on ice and homogenised using either a bead mill or pellet pestle. For the bead mill, eight 2.3mm beads (#11079125z BioSpec Products Inc.) and approximately twenty-five 1.0mm beads (#11079110z BioSpec Products Inc.), 1ml of ice cold TRIzol and thawed tissue sample were added to a 2ml centrifuge tube. Samples were homogenised using a Retsch Mixer Mill MM300 for two minutes at 30.0/s, with all manifolds/adaptors pre-cooled on ice. For the pellet pestle, tissue samples were transferred to a 2ml U-bottom shaped centrifuge tube containing 150µl Trizol and homogenised with Kimble chase cordless motor and polypropylene pellet pestle (Sigma) for 3-4min or until the sample was completely dispersed. Additional Trizol was added for a final volume of 1ml and the sample was passed through a 19 gauge needle 15 times to ensure complete lysis of cells. Samples were assessed for RNA quantity and integrity using the Nanodrop1000 and Agilent Bio-analyser, respectively. The RNA quantity and sample integrity using the pellet pestle was superior to the bead mill (S1 table) and was selected as the optimal method for RNA extraction from fungiform papillae.

**S1 Table.** **Comparison of sample homogenisation from fungiform papillae.**

| Method | Sample | Quantity (ng/µl) | Integrity (RIN) |
| --- | --- | --- | --- |
| Bead mill | 1 | 16.6 | 2.7 |
|  | 2 | 17.1 | 2.6 |
| Pellet pestle | 1 | 43.9 | 9.6 |
|  | 2 | 56.6 | 9.8 |
